# Integrated Performance Evaluation of the Smart Body Area Networks Physical Layer for Future Medical and Healthcare IoT †

**DOI:** 10.3390/s19010030

**Published:** 2018-12-21

**Authors:** Kento Takabayashi, Hirokazu Tanaka, Katsumi Sakakibara

**Affiliations:** 1 Department of Information and Communication Engineering, Faculty of Computer Science and Systems Engineering, Okayama Prefectural University, 111 Kuboki, Soja, Okayama 719-1197, Japan; sakaki@c.oka-pu.ac.jp; 2 Graduate School of Information Sciences, Hiroshima City University, 3-4-1, Ozuka-Higashi, Asa-Minami-Ku, Hiroshima 731-3194, Japan; hi.tanaka@m.ieice.org

**Keywords:** ETSI SmartBAN, Medical and healthcare IoT, Preamble detection, Physical layer, PN sequence, Integrated evaluation

## Abstract

This paper performs integrated performance evaluation, including preamble detection in the Smart Body Area Networks (SmartBAN) physical layer (PHY). The system specifications for a PHY and media access control layer (MAC) in SmartBAN, which is a standard for medical and health care advanced by the European Telecommunications Standards Institute (ETSI), were issued in April 2015. In the PHY, the packet structure has a two-octet preamble, which is used, e.g., for timing synchronization. However, it is considered that the current preamble structure is not appropriate for handling medical and healthcare data that are required to have high reliability because of the too simple structure. Therefore, we propose adding a start frame delimiter (SFD) to correctly detect the header position. Computer simulations indicate that preambles with an SFD consisting of an orthogonal maximal length sequence (M-sequence) perform better than SmartBAN and similar approaches, particularly when transmitting over the IEEE model CM3. In addition, the packet error ratio (PER) and energy efficiency are evaluated in an integrated manner while taking preamble detection into consideration. The numerical results from computer simulations indicated the best performance with respect to PER was achieved using a preamble with orthogonal M-sequences of 4 octets. However, for energy efficiency, better results were obtained using a preamble with orthogonal M-sequences of 2 octets. Additionally, the theoretical analysis found the optimum length of the PHY packet to achieve the maximum energy efficiency with PER less than 10^−2^.

## 1. Introduction

**Figure 1:**
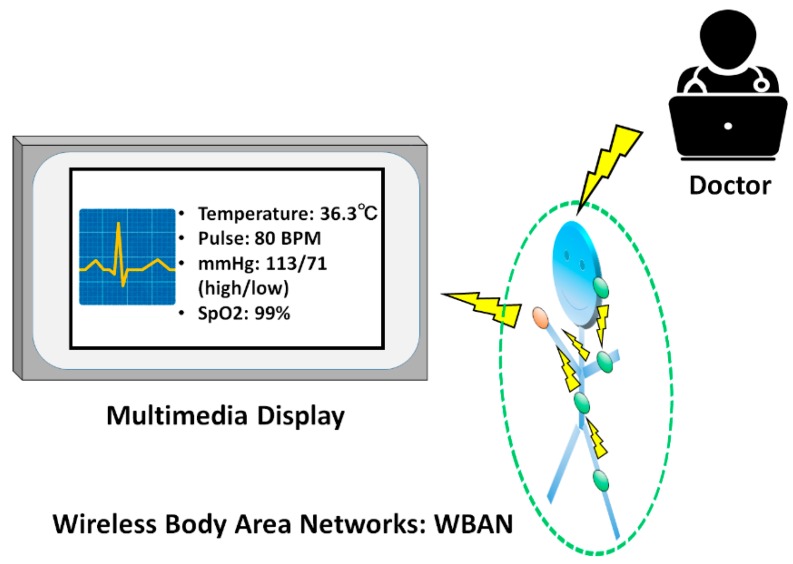
Example of a medical and healthcare system using WBAN.

In recent years, a medical and healthcare Internet of Things (IoT) system has attracted attention as a means to support home medical care or a remote medical care. The system involves wearable wireless vital sign sensors or medical robots (Figure 1) [1,2]. A TV-based medical service (t-health) for supportive living is proposed, which enables elderly people and physically handicapped people to easily collect basic parameter values and send them to a remote monitoring center [1]. The authors design, implement and test the solution that provides social health services to elderly people at home based on access to smart TV technology and all services [2]. Then, wireless body area networks (WBAN) are well-known medical and healthcare IoT systems [3,4,5,6,7]. WBAN consist of a collection of low-power, miniaturized, invasive or non-invasive lightweight devices with wireless communication capabilities that operate near the human body. These devices are placed in, on or around the body and monitor vital information [3,4,5,6,7]. For example, the overview of WBAN, and the recent technical and design challenges are introduced [3,4,5,6]. Comprehensive research and detailed analysis of coexistence problems and interference mitigation solutions in the WBAN are provided [6]. The introduction of cloud support type WBAN and the main issues to be addressed for its development and management are described [7]. By the way, IEEE 802.15.6 (Institute of Electrical and Electronics Engineers, New York, NY, USA) which is a system standard was issued in 2012 [8]. Subsequently, system specifications for a physical layer (PHY) and a media access control layer (MAC) in smart body area networks (SmartBAN) were issued in April 2015. These specifications represent a standard for medical and other health care advanced by the European Telecommunications Standards Institute (ETSI) [9,10].

SmartBAN characteristics include the following. Firstly, a star-type topology is adopted, in which the hub collects data measured by the node and sends the collected data to external equipment. Secondly, complex specifications (in particular, the MAC layer), which were a problem in IEEE 802.15.6, are simplified as much as possible. Thirdly, the SmartBAN standard focuses on transmission and reception of emergency signals with low delay not considered in existing wireless communication systems. In addition, SmartBAN is standardized to satisfy the following technical requirements:

1. Ultra-low power consumption
2. Coexistence with other systems
3. Optimum control of QoS.

Evaluation and testing of SmartBAN have mainly focused on the MAC protocol [11,12,13,14,15]. For example, [12,14] derived closed-form analytical models for the uplink transmission delay and presented an optimal inter-beacon interval frame for SmartBAN to reduce delay or energy consumption. In addition, the same authors [12,13,14] evaluated downlink delay performance based on SmartBAN and improved downlink delay to adopt fixed-length exhaustive transmission [13]. However, the SmartBAN PHY has not been sufficiently discussed.

Previously, we provided performance evaluations of an error-control scheme in the SmartBAN PHY under several conditions [16]. In particular, we evaluated performance when Bose–Chaudhuri–Hocquenghem (BCH) codes with nearly the same redundancy as the packet repetition were applied and then compared this performance with that of the standard scheme. In addition, retransmission performance was evaluated. Numerical results indicated that retransmission substantially improved the packet error ratio and energy efficiency of the IEEE model CM3 which is a channel model of wearable WBAN [16,17].

In this study, first, preamble detection in the SmartBAN PHY is evaluated because other studies on the ETSI SmartBAN PHY, including our previous study, do not fully consider this point [16,18,19,20,21,22,23,24,25]. Additionally, we propose a preamble structure to which a start frame delimiter (SFD) is added to correctly detect the header position under not only the additive white Gaussian noise (AWGN) channel (the ideal environment) but also the IEEE model CM3 (close to real environment). Several SFD candidates were selected, and their preamble detection performances are evaluated by computer simulations. The best performance is obtained when an orthogonal maximal length sequence (M-sequence) is used as the SFD under the AWGN channel and IEEE model CM 3.

As the second main contribution, we also provide a novel integrated performance evaluation of the packet error ratio (PER) and energy efficiency while taking into account preamble detection in the SmartBAN PHY. Novel numerical results by computer simulations indicate that the best performance with respect to PER is when a preamble with orthogonal M-sequences of 4 octets is used. However, for energy efficiency, better results are obtained using a preamble with orthogonal M sequences of 2 octets. Furthermore, the optimum length of the PHY packet to achieve the maximum energy efficiency with PER less than 10^−2^ is found by the theoretical analysis which was not conducted in previous work. Those results suggest that it is better to change the length of SFD according to channel conditions and the payload size because the maximum energy efficiency is affected by the overall packet size. They are also expected to contribute to the design of highly reliable and energy-efficient SmartBAN.

The remainder of this paper is organized as follows. In Section 2, we summarize the SmartBAN PHY. In Section 3, our proposed preamble structure and its performance are explained. The numerical results of an integrated performance evaluation are provided in Section 4. Conclusions and suggestions for future research are presented in Section 5.

## 2. Summary of the SmartBAN PHY

### 2.1. Frequency Spectrum

**Figure 2:**
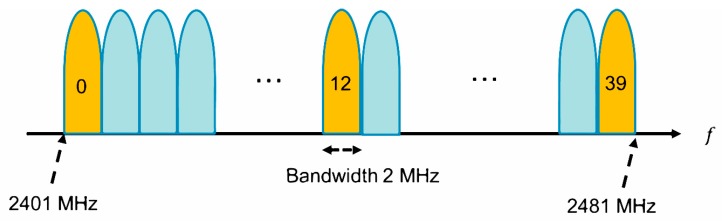
SmartBAN frequency spectrum.

In SmartBAN, a frequency band of 2401 MHz to 2481 MHz is used, and each channel has a bandwidth of 2 MHz (Figure 2). In addition, each center frequency is defined by the following equation:

(1) $$f_{c}=2402+2n\;\mathrm{MHz},\;\mathrm{for}\;n=0\;\mathrm{to}\;39$$

Here, *n* is the channel number.

### 2.2. Packet Structure

**Figure 3:**
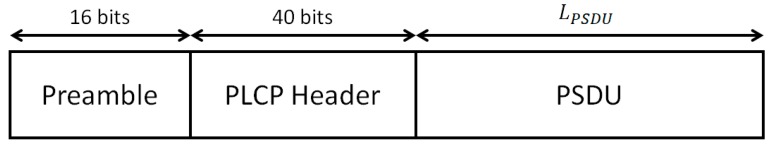
PPDU structure.

Figure 3 shows the structure of a packet in the physical layer. The physical-layer protocol data unit (PPDU) has a sixteen-bit preamble “1010101010101010” used for frequency synchronization, timing synchronization, and automatic gain control. The physical layer convergence protocol (PLCP) header consists of the packet length, the PHY scheme and other components. It is encoded by the (36, 22) shortened BCH code as an error-correcting code and CRC-4-ITU as an error-detecting code. The physical-layer service data unit (PSDU) is either an encoded or un-coded MAC protocol data unit (MPDU) [9,10]. The MPDU is encoded by CRC-8(-CCITT) and CRC-16(-CCITT) as an error-detecting code.

### 2.3. Modulation and Error Controlling

**Figure 4:**
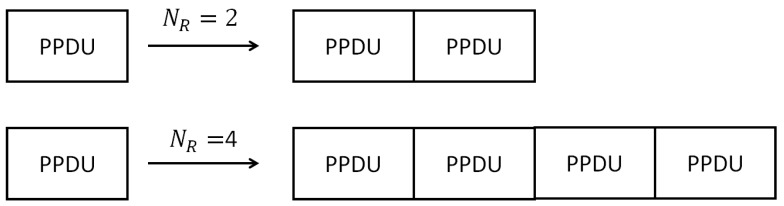
PPDU repetition.

In the SmartBAN PHY, Gaussian frequency shift keying (GSFK) with a bandwidth-bit period product BT = 0.5 and modulation index h = 0.5 is used as a modulation scheme. However, as an error control scheme in the SmartBAN PHY, we may use a scheme of repeatedly transmitting PPDUs and a scheme encoding the MPDU by using the (127, 113) BCH code as an error-correcting code. In the method of repeated transmission (Figure 4), it is possible to set the number of repetitions: $N_{R}$ = 2, 4. For the (127, 113) BCH encoding, the following generator polynomial is used:

(2) $$g\left(x\right)=x^{14}+x^{9}+x^{8}+x^{6}+x^{5}+x^{4}+x^{2}+x+1$$

Then, the (36, 22) shortened BCH code used in the PLCP header is generated based on the (127, 113) BCH code. It is also possible to use these schemes in combination. Table 1 summarizes the throughput in the PHY.

## 3. Modified Preamble Structure and System Model

### 3.1. Proposed Packet Structure

We propose modifying the preamble structure in ETSI SmartBAN. The main reason for the motivation is that the current preamble structure is too simple to perform highly reliable communication dealing with medical-healthcare information. Hence, if two bits of ‘10’ are detected before and after the preamble by noise, fading and so on, detection of the beginning of the PLCP header fails easily. That is, packet errors occur frequently, resulting in a decrease in energy efficiency.

**Figure 5:**
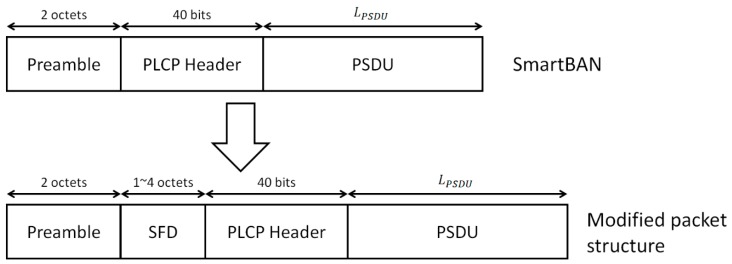
Proposed packet structure. The top section of the figure shows the SmartBAN packet structure. The bottom section shows the proposed structure.

To correctly detect the position of the PLCP header, an SFD is added between the two-octet preamble and the PLCP header (Figure 5). When detecting the header position, cross-correlation is performed on the known modulated SFD symbol. The advantage of this approach is that it can be realized without changing the standards drastically. Thus, the two-octet preamble component can also play a conventional role.

### 3.2. System Model

**Figure 6:**
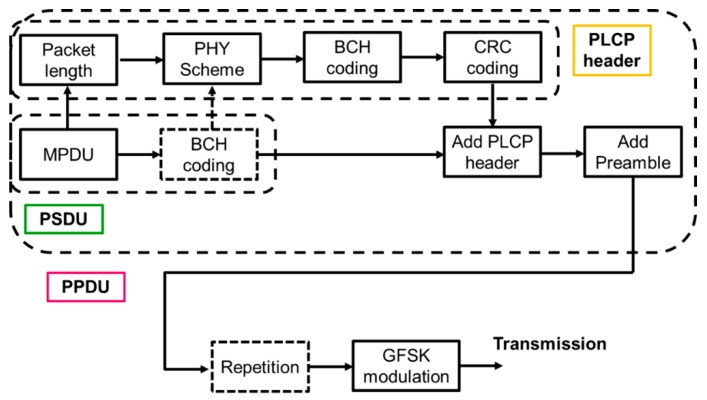
Block diagram of PHY transmitter.

**Figure 7:**
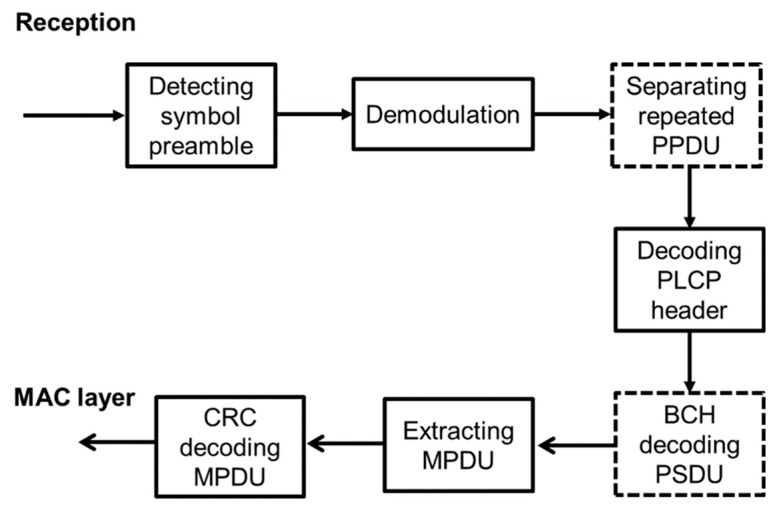
Block diagram of PHY receiver.

Figure 6 and Figure 7 show the system model of this study. Figure 6 presents a block diagram of PHY packet generation at the transmitter, and Figure 7 presents a block diagram of the method used to extract the MPDU from the PHY packet at the receiver. The dotted box indicates an optional operation. At the transmitter, the information of the MPDU (the length, BCH coding, etc.) is entered in the PLCP header. After BCH coding and CRC coding are performed on the PLCP header, the MPDU, the PLCP header, and the preamble including SFD are concatenated to create the PPDU. The PPDU is modulated with GFSK, and then transmitted to the receiver. For receiving, the symbol preamble detection is performed from the modulated PPDU, and the head of the PCLP header is determined. Thereafter, the PPDU is demodulated, and the PCLP header is decoded. If bit errors are not detected, the MPDU is extracted and CRC decoding is performed on it. If a bit error is detected in the PLCP header or the MPDU, negative-acknowledgement (NACK) is sent back to the sender to prompt retransmission. In this study, we consider cases of non-BCH encoding, (127.113) BCH encoding, and (127.63) BCH encoding as our proposed option for the MPDU based on the results of our previous study [16]. Therefore, PPDU repetition is not considered.

Previous researches were mainly evaluated on the performance of the PLCP header and the PSDU. In other words, it was assumed that preamble detection was ideal in them. However, the SmartBAN PHY cannot be evaluated correctly unless characteristics of preamble detection are considered. Another motivation for this work is to find the SFD that maximize the performance of the SmartBAN PHY.

## 4. Integrated Performance Evaluation

### 4.1. Simulation Parameter

In this section, an integrated performance evaluation of the PER and energy efficiency considering preamble detection in the SmartBAN PHY is discussed. The energy efficiency parameters were adopted from the literature (41–46). Thus, energy efficiency is derived as follows:

(3) $$\eta \equiv \frac{P_{succ}L_{info}}{E_{link}}$$

Here, $E_{link}$ is the energy consumption of the communication link and $P_{succ}$ is the transmission success ratio. $P_{succ}$ can be expressed as follows:

(4) $$P_{succ}=1-PER=\left(1-P_{fail,preamble}\right)\left(1-P_{e,PLCPheader}\right)\left(1-P_{e,PSDU}\right)$$

Here, PER is the packet error ratio, $P_{fail,preamble}$ is the failure preamble detection ratio, $P_{e,PLCPheader}$ is the PLCP header error ratio, and $P_{e,PSDU}$ is the PSDU error ratio. In addition, because retransmission is not considered in this computer simulation, $E_{link}$ can be simply described as follows:

(5) $$E_{link}=\left(L_{preamble}+L_{SFD}+L_{PLCPheader}+L_{PSDU}\right)\left(P_{tx}+P_{rx}\right)/R_{sym}+\left(\varepsilon_{enc}+\varepsilon_{dec}\right)$$

Here, $R_{sym}$ is the symbol rate; $L_{preamble}$, $L_{SFD}$, $L_{PLCPheader}$, and $L_{PSDU}$ are the length of the preamble, SFD, PLCP header, and PSDU, respectively; $P_{tx}$ and $P_{rx}$ are transmitter and receiver power consumption, respectively; and $\varepsilon_{enc}$ and $\varepsilon_{dec}$ are encoding and decoding energies, respectively [26,27,28,29,30,31].

In this section, we describe performance evaluation by computer simulations of preamble detection in the SmartBAN PHY and our proposed method. The main parameters of the computer simulations are listed in Table 2. The computer simulator was constructed by MATLAB. Then, the “comm. Preamble Detector System object” in MATLAB was used for preamble detection, and the detection threshold was set to the length of each SFD minus one ($L_{SFD}-1$). The entire two-octet preamble was only correlated for SmartBAN, and the threshold was set to the length of the preamble minus one ($L_{preamble}-1$). Table 3 summarizes the SFDs used in the computer simulations. The reason for choosing these sequences is that they can be handled in units of octets. In this computer simulation, the AWGN channel and IEEE model CM3, which is the channel model of wearable WBAN, were used [17]. Similarly, IEEE model CM3 was applied to the path loss model. The path loss model of IEEE model CM 3 when using the 2.4 GHz band is shown in Table 4 and (6):

(6) $$PL\left(d\right)=a\times log_{10}d+b+N$$

Here, a and b are coefficients of linear fitting, d is the Tx-Rx distance in mm, and N is a normally distributed variable with standard deviation $\sigma_{N}$. The K factor in decibels ($K_{dB}$) and each parameter when assuming frequency flat fading are as shown in Table 5 and (7):

(7) $$K_{dB}=K_{0}-m_{k}PL\left(d\right)+\sigma_{k}n_{k}$$

The meaning of each parameter is defined in [17]. $K_{0}$ is the fit with measurement data for the *K* factor for low path loss, $m_{k}$ is the slope of the linear correlation between path loss and *K* factor, $\sigma_{k}$ is the log-normal variance of the measured data between path loss and *K* factor, and $n_{k}$ is zero mean and unit variance Gaussian random variable. From (7), we can observe that $K_{dB}$ decreases as the path loss increases.

### 4.2. Results of Preamble Detection

**Figure 8:**
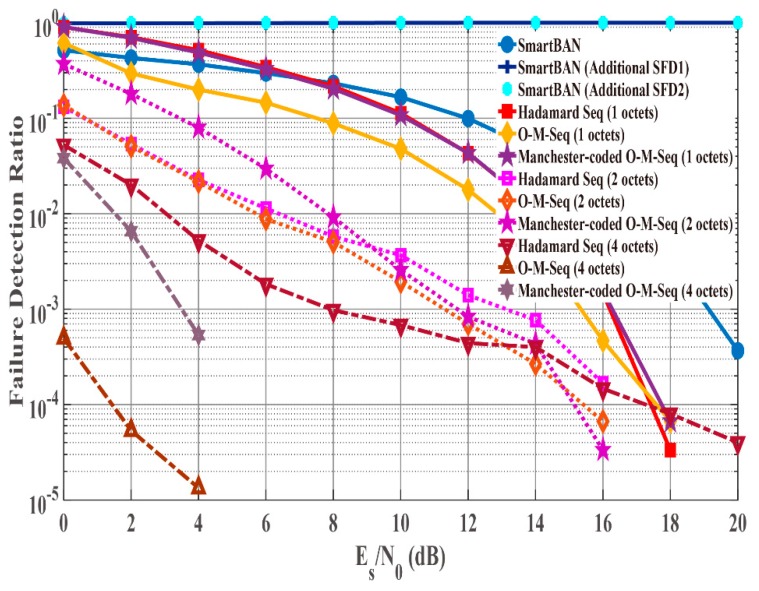
Failure detection ratio under the AWGN channel as function of $E_{s}/N_{0}$.

**Figure 9:**
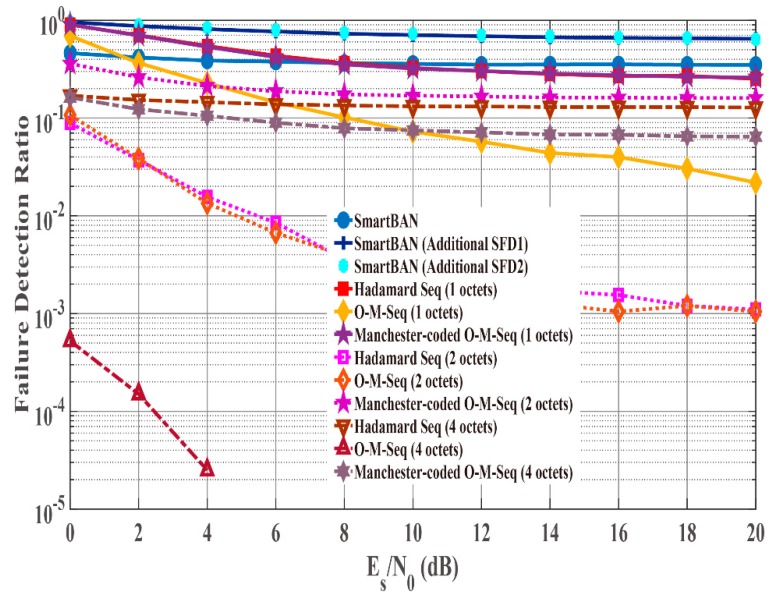
Failure detection ratio under IEEE model CM3 as function of $E_{s}/N_{0}$ (*K* factor = 4.5 ).

**Figure 10:**
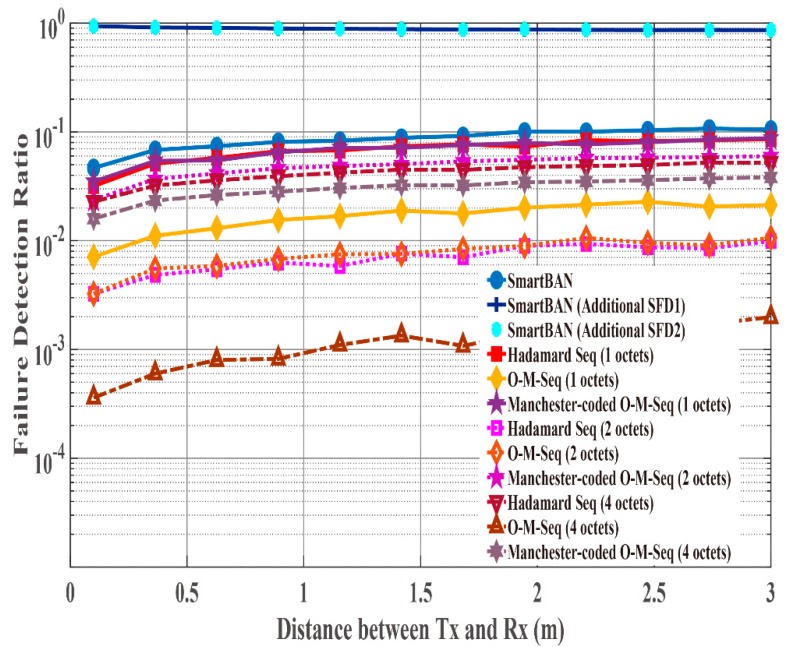
Failure detection ratio under IEEE model CM3 as a function of communication distance.

**Figure 11:**
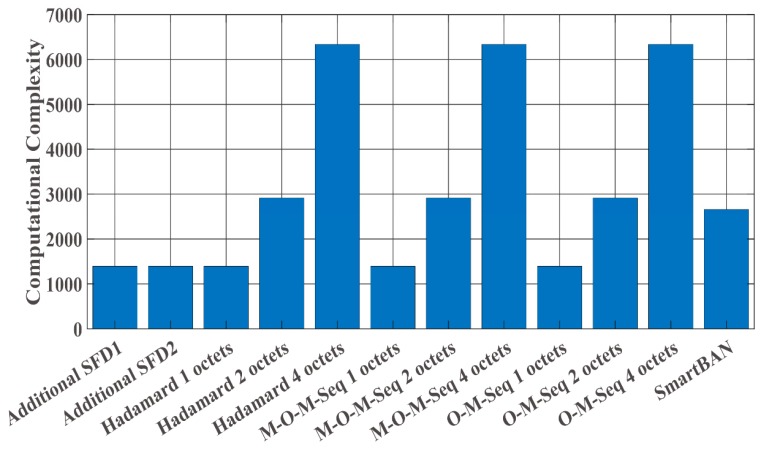
Computational complexity for preamble detection.

Figure 8 and Figure 9 show failure detection ratio performance under the AWGN channel and IEEE model CM3, respectively, as a function of energy per symbol to noise power spectral density ($E_{s}/N_{0}$). From (4), the *K* factor in true value = 4.5 when *d* ≈ 1.0 m (Figure 9). In addition, Figure 10 shows that under IEEE model CM3 the failure detection ratio is a function of the communication distance (d) between a transmitter and a receiver. Here, “failure detection” includes false detection and non-detection. False detection means to detect the erroneous position as the correct PLCP header position. In contrast, non-detection means to fail to detect peaks by cross-correlation. As shown in the figures, the SmartBAN preamble exhibits good performance under the AWGN channel and good $E_{s}/N_{0}$ conditions. On the other hand, its performance was bad under IEEE model CM3. The reason is that the auto-correlation characteristic is not good, and the cross-correlation peak did not exceed the threshold due to large noise or fading. From Figure 8, Figure 9 and Figure 10, additional SFD 1 and 2 were unable to correctly detect each preamble. This is because they were highly similar to part of the two-octet preamble. Of the other sequences, the orthogonal M-sequences performed better. In particular, the four-octet orthogonal M-sequence obtained good results even in very poor channel conditions. The reason for this outcome is considered to be that the auto-correlation characteristic is higher than other sequences. Table 6, Table 7 and Table 8 summarize $E_{s}/N_{0}$ and d when failure detection ratio satisfies $10^{-2}$ or less under the AWGN channel and IEEE model CM3. The reason is that one of the criteria for PER to be satisfied is $10^{-2}$ [3,4,5,6,7,8]. Under the AWGN channel, the SmartBAN preamble and SFDs other than additional SFD 1 and 2 satisfy this condition. Basically, the longer the length of the SFD is, the lower $E_{s}/N_{0}$ is satisfying it. As shown in Table 7 and Table 8, the two-octet Hadamard sequence, the two-octet and the four-octet orthogonal M-sequence can only satisfy the condition. The reason that the two-octets Hadamard sequence satisfies it is thought to be because its randomness is relatively high. Finally, Figure 11 presents a comparison for computational complexity required for preamble detection. Here, computational complexity means the amount of multiplication until correct preamble detection is performed. The longer the sequence used for the correlation, the more the computational complexity increases. The computational complexity of one-octet SFDs is more than four times larger than that of four-octets SFDs. Also, the computational complexity in SmartBAN is somewhat less than that in two-octet SFDs by the length of SFD. That is, there is a trade-off relationship between the computational complexity and the preamble detection capability. On the other hand, it can be seen from Figure 9 and Figure 10 that it is necessary to use two or more octets SFD to satisfy the failure detection ratio of $10^{-2}$ or less.

### 4.3. Results of Integrated Performance Evalution

**Figure 12:**
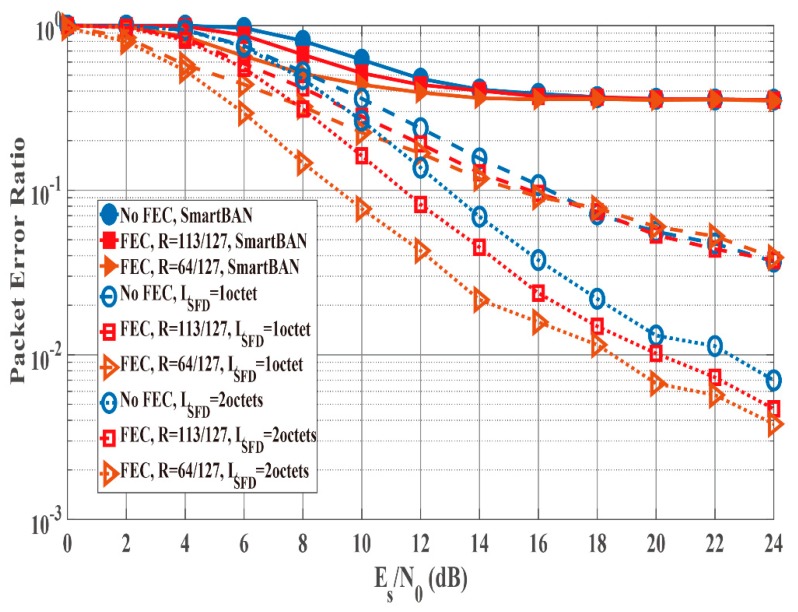
PER performance as a function of $E_{s}/N_{0}$ (K factor = 4.5). Cases of without and with an SFD ($L_{SFD}=$ 1 octet and 2 octets).

**Figure 13:**
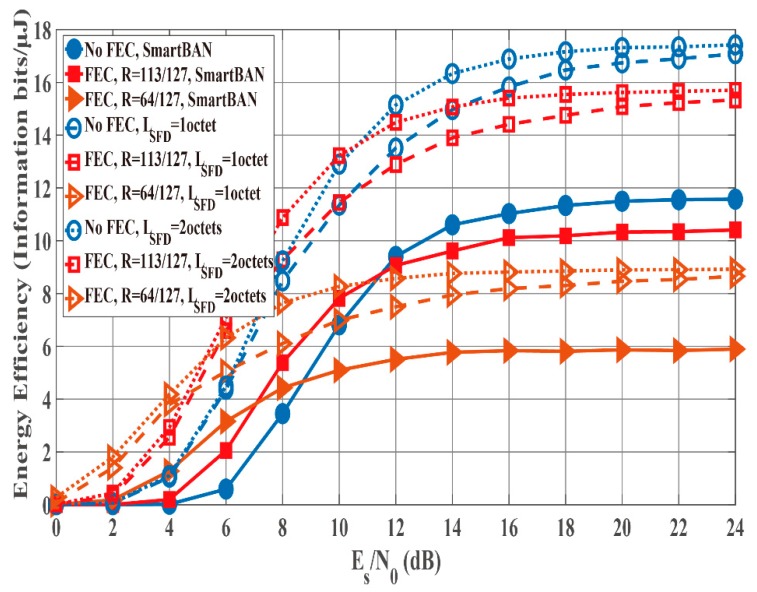
Energy efficiency performance as a function of $E_{s}/N_{0}$ (K factor = 4.5). Cases without and with an SFD ($L_{SFD}=$ 1 octet and 2 octets).

Figure 12 and Figure 13 show the PER and energy efficiency performances as a function of $E_{s}/N_{0}$ when the K factor’s true value = 4.5. Regarding the PER shown in Figure 12, BCH encoding, particularly the (127, 64) BCH code, performs better than no BCH encoding. In addition, we assume that the orthogonal M-sequences are used as the SFD because they perform better than other sequences. Regarding the influence of preamble detection, the SmartBAN preamble performs worse than when an SFD is used. In addition, the PER converges to approximately 0.3 as $E_{s}/N_{0}$ increases because of $P_{fail,preamble}$ of the SmartBAN preamble. However, the PER improves as $L_{SFD}$ becomes longer. The case with an SFD whose $L_{SFD}$ = 2 octets obtains over 3 dB more than the case with an SFD whose $L_{SFD}$ = 1 octet. In addition, the case with a two-octet SFD achieves PER $=10^{-2}$ when $E_{s}/N_{0}>18$ dB. As shown in Figure 13, no encoding has larger energy efficiency under high SNR conditions because of the small redundancy, while BCH encoding has larger energy efficiency under low SNR conditions because of the smaller $P_{e,PSDU}$ Thus, energy efficiency without an SFD is more than 30% less than with an SFD under high SNR conditions although an overhead is added in the case with an SFD. This is because $P_{fail,preamble}$ of the SmartBAN preamble is much larger than that of the case with an SFD.

**Figure 14:**
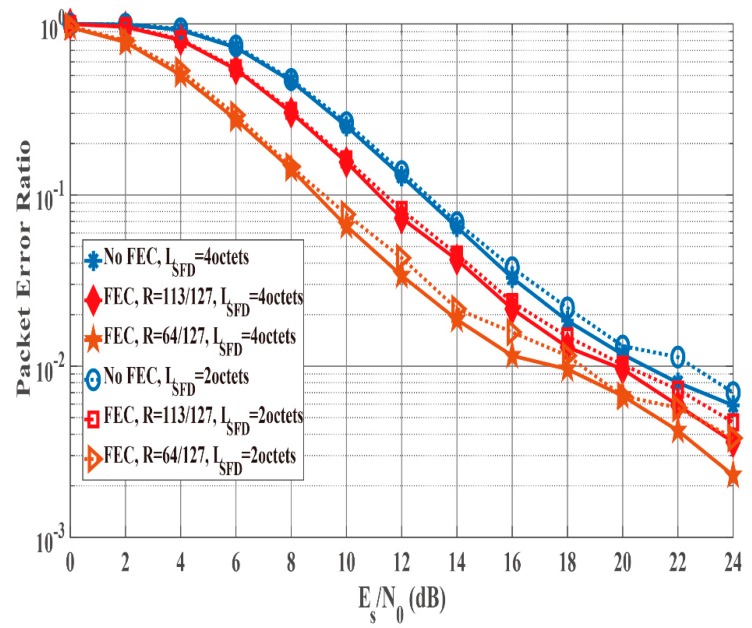
PER performance as a function of $E_{s}/N_{0}$ (K factor = 4.5). Case with an SFD ($L_{SFD}=$ 2 octets and 4 octets).

**Figure 15:**
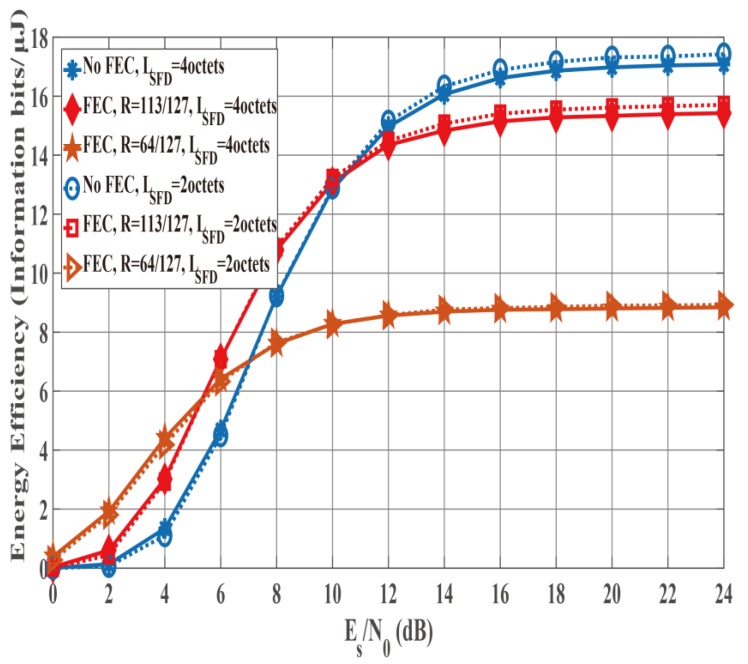
Energy efficiency performance as a function of $E_{s}/N_{0}$ (*K* factor = 4.5). Case with an SFD (*L_SFD_* = 2 octets and 4 octets).

Figure 14 and Figure 15 compare the PER and the energy efficiency performances of the case with SFD whose $L_{SFD}=$ 2 octets to those of the case with SFD whose $L_{SFD}=$ 4 octets as a function of $E_{s}/N_{0}$ in the case that K factor’s true value = 4.5. As shown in Figure 14, the PER of the case with a two-octet SFD is similar to that of the case with an SFD whose $L_{SFD}=$ 4 octets. Therefore, $P_{e,PSDU}$ can be said to be dominant under these conditions. However, Figure 15 shows that the energy efficiency of the case with an SFD whose $L_{SFD}=$ 2 is somewhat better. The reason is that the case with an SFD whose $L_{SFD}=$ 2 has a smaller overhead, whereas the PER is nearly the same as that of the case with an SFD whose $L_{SFD}=$ 4, as in Figure 14.

**Figure 16:**
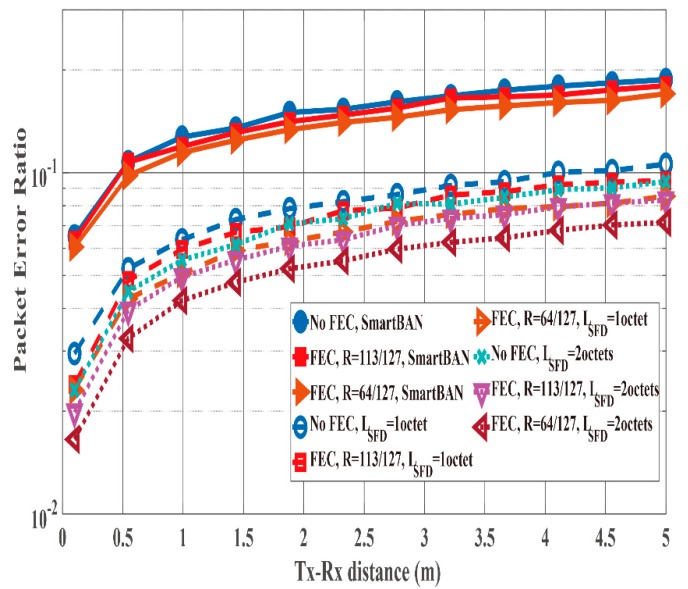
PER performance as a function of the Tx-Rx distance. Cases without and with SFDs (*L_SFD_* = 1 octet and 2 octets).

**Figure 17:**
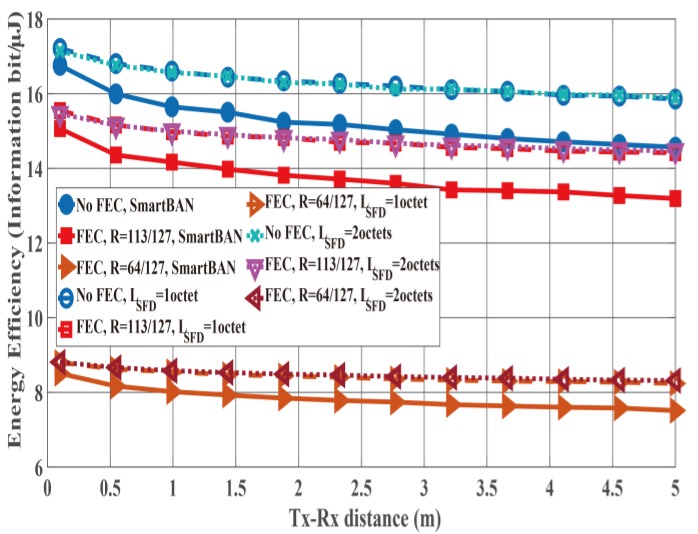
Energy efficiency performance as a function of the Tx-Rx distance. Cases without and with SFDs (*L_SFD_* = 1 octet and 2 octets).

Figure 16 and Figure 17 show the PER and energy efficiency performances as a function of the Tx-Rx distance. As presented in Figure 16, the case with no SFD case exhibits worse PER performance than the cases with an SFD because of the large $P_{fail,preamble}$. In particular, the Tx-Rx distance must be less than 50 cm to satisfy the PER of the case without an SFD = $10^{-1}$. However, the cases of SFDs satisfy PER $\leq 10^{-1}$ when the Tx-Rx distance is less than 4 m. Thus, the energy efficiency of the cases with SFDs is better than that of the case without an SFD (Figure 17) due to the large PER of the no SFD case. In addition, the case with an SFD whose $L_{SFD}=$ 1 octet achieves nearly the same energy efficiency as the case with an SFD whose $L_{SFD}$ 2 octets even though the former’s PER is larger than that of the latter. This is because the overhead of the former is smaller than that of the latter.

**Figure 18:**
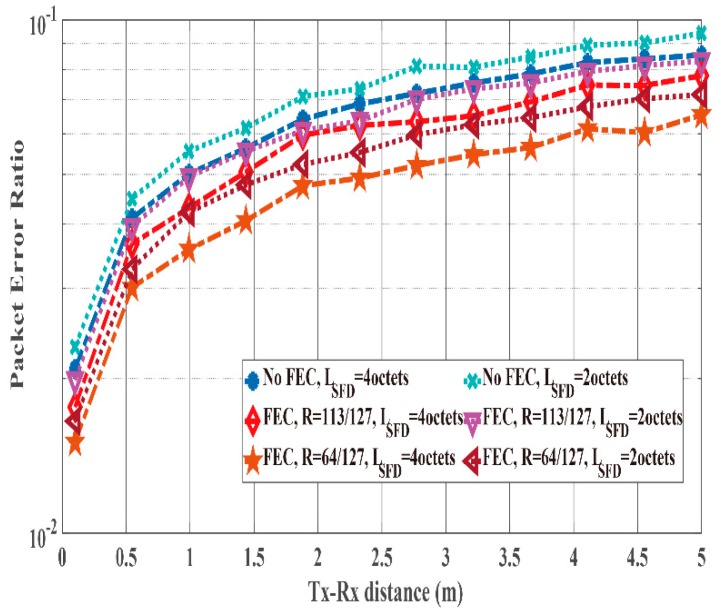
PER performance as a function of the Tx-Rx distance. Case with an SFD (*L_SFD_* = 2 octets and 4 octets).

**Figure 19:**
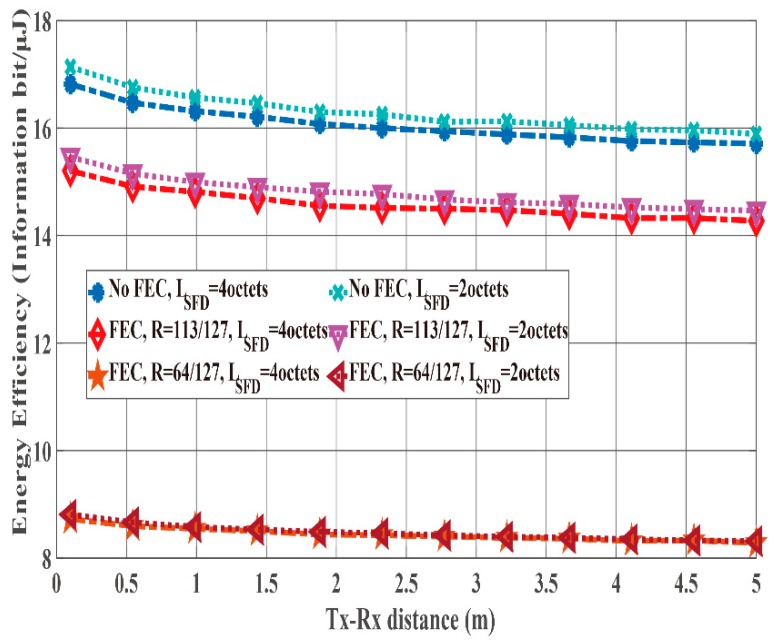
Energy efficiency performance as a function of the Tx-Rx distance. Case with an SFD (*L_SFD_* = 2 octets and 4 octets).

Figure 18 and Figure 19 compare the PER and the energy efficiency performances of the case with an SFD whose $L_{SFD}=$ 2 octets to those of the case with an SFD whose $L_{SFD}=$ 4 octets as a function of the Tx-Rx distance. Figure 18 shows that the PER of the case with an SFD whose $L_{SFD}=$ 4 octets is better than that of the case with an SFD whose $L_{SFD}=$ 2 octets because $P_{fail,preamble}$ of the SFD whose $L_{SFD}=$ 2 octets has an influence on the PER (Figure 9). However, the energy efficiency of the case with an SFD whose $L_{SFD}=$ 2 octets is better than that of the case with an SFD whose $L_{SFD}=$ 4 octets except the case with (127, 63) BCH encoding (Figure 19). The reason is that the overhead of the latter has a large influence on energy efficiency.

According to the computer simulations, the case with an SFD whose *L_SFD_* = 4 octets exhibits the best performance for PER, while the case with an SFD whose *L_SFD_* = 2 octets achieves the best energy efficiency.

## 5. Discussion about Optimum *L_PSDU_*

In the previous section, computer simulations were performed with the fixed un-coded $L_{PSDU}$. In this section, the optimum un-coded $L_{PSDU}$ that satisfies a certain condition is discussed. Here it is assumed that the maximum energy efficiency with PER less than $10^{-2}$ is achieved as a condition.

**Figure 20:**
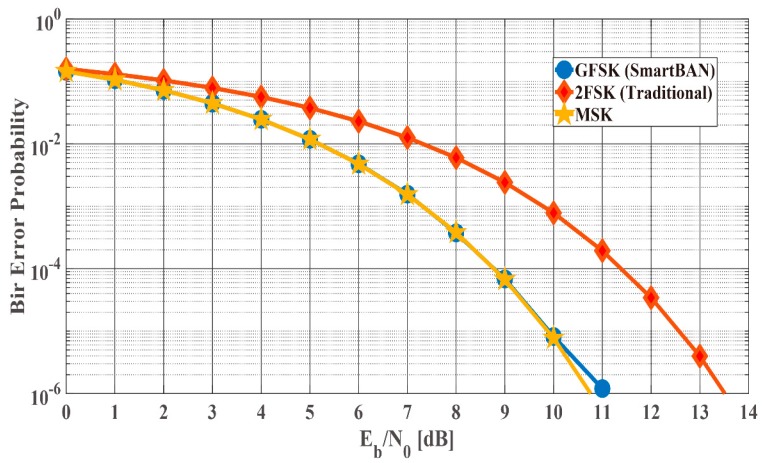
Bit error probability under the AWGN channel as a function of $E_{s}/N_{0}$. GFSK used in SmartBAN, minimum-shift keying (MSK) and 2FSK are compared.

First of all, Figure 20 shows the bit error probability ($P_{b}$) of the GFSK used in SmartBAN, minimum-shift keying (MSK) and 2FSK under the AWGN channel as a function of $E_{s}/N_{0}$. As show in Figure 20, the bit error probability of the GFSK used in SmartBAN is almost the same as that of MSK. The $P_{b}$ of MSK under the AWGN channel is expressed as follows [34]:

(8) $$P_{b}=2Q\left(\sqrt{\frac{2E_{s}}{N_{0}}}\right)-2Q^{2}\left(\sqrt{\frac{2E_{s}}{N_{0}}}\right)$$

Here, Q(·) is the Q function. Also, the $P_{b}$ of MSK under the Rician fading channel is expressed as follows [34]:

(9) $$P_{b}=\frac{2}{\pi }\overset{\frac{\pi }{2}}{\underset{0}{\int }}M_{\gamma }\left(-\frac{1}{\sin^{2}\theta }\right)d\theta -\frac{2}{\pi }\overset{\frac{\pi }{4}}{\underset{0}{\int }}M_{\gamma }\left(-\frac{1}{\sin^{2}\theta }\right)d\theta$$

(10) $$M_{\gamma }\left(s\right)=\frac{1+K}{1+K-s\bar{\gamma }}e^{\left[\frac{Ks\bar{\gamma }}{1+K-s\bar{\gamma }}\right]}$$

where K is the K factor (linear scale), and $\bar{\gamma }$ is the average $E_{s}/N_{0}$. In this discussion, it is assumed that (9) is $P_{b}$ of GFSK used in the SmartBAN PHY under the IEEE model CM3.

Then, $P_{e,PSDU}$ and $P_{e,PLCPheader}$ are expressed as follows [29]:

(11) $$P_{e,PLCPheader}=P_{e,PSDU}=1-{\left(1-P_{block}\right)}^{\left\lceil \frac{L_{PSDU\;or\;PLCPheader}}{N_{code}}\right\rceil }$$

(12) $$P_{block}\leq \overset{N_{code}}{\underset{h=t+1}{\sum }}\left(\begin{array}{c} N_{code}\\ h \end{array}\right)P_{b}{}^{h}{\left(1-P_{b}\right)}^{N_{code}-h}$$

Here $N_{code}$ is the code-length of BCH code, and t is the number of correctable bits in the block. In the non-BCH coding case, $N_{code}=1$ and t=0. Then, the $E_{s}/N_{0}$ at a receiver can also be expressed by using PL(d) and parameters listed in Table 2 as follows:

(13) $${\left(E_{s}/N_{0}\right)}_{dB}=P_{r}-P_{n}$$

(14) $$P_{r}=P_{t}-PL\left(d\right)$$

(15) $$P_{n}=N_{0}\left(BW\right)+{\left(NF\right)}_{dB}+I_{dB}$$

**Figure 21:**
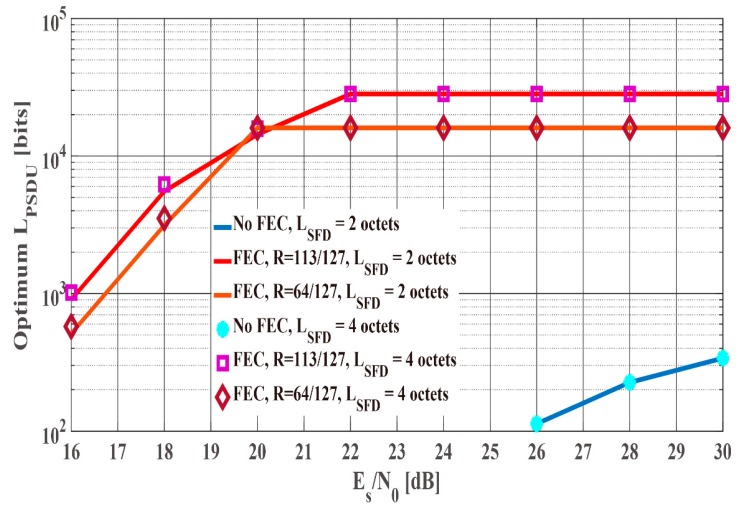
The optimum $L_{PSDU}$ under the IEEE model CM3 as a function of $E_{s}/N_{0}$.

**Figure 22:**
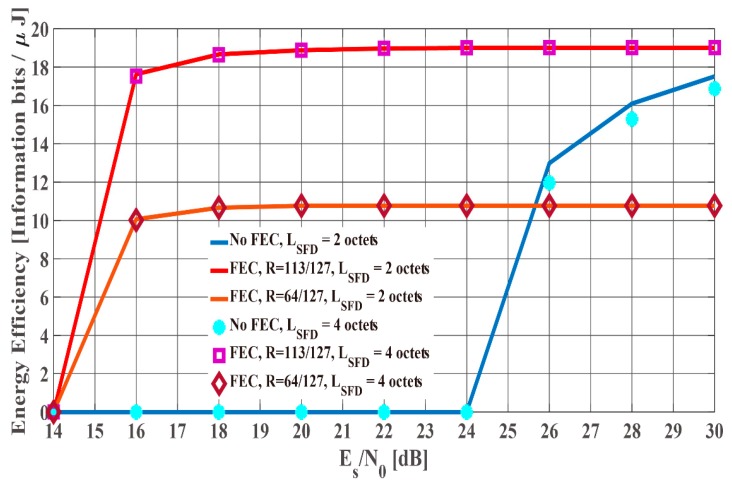
The maximum energy efficiency under the IEEE model CM3 as a function of $E_{s}/N_{0}$.

By using (3)–(7) and (9)–(15), the optimum $L_{PSDU}$ that satisfies the above condition is searched for in the full search. Here, the results of the two-octet and the four-octet orthogonal M-sequences obtained by computer simulation under the IEEE model CM3 are used as for $P_{fail,preamble}$. Then, $L_{PSDU}$ is searched for multiples of 113 (no-BCH encoding case and (127,113) BCH encoding case) and 64 ((127,64) BCH encoding case) because of the information bit length of each BCH codes. In addition, the maximum $L_{PSDU}$ is set to $2^{15}-1$ in accordance with the SmartBAN specification. Figure 21 and Figure 22 present the optimum $L_{PSDU}$ and the maximum energy efficiency under the IEEE model CM3 as a function of $E_{s}/N_{0}$. As show in Figure 21, the optimum $L_{PSDU}$ of the four-octet SFD is slightly larger than that of the two-octet SFD in the cases of BCH encoding under less than 20 dB $E_{s}/N_{0}$ conditions because of each $P_{fail,preamble}$. On the other hand, the maximum energy efficiency of the two-octet SFD is larger than that of the four-octet SFD in the no BCH encoding case under over 26 dB $E_{s}/N_{0}$ conditions in Figure 22. This is because each optimum $L_{PSDU}$ is short, that is, it is more susceptible to the length of the SFD.

**Figure 23:**
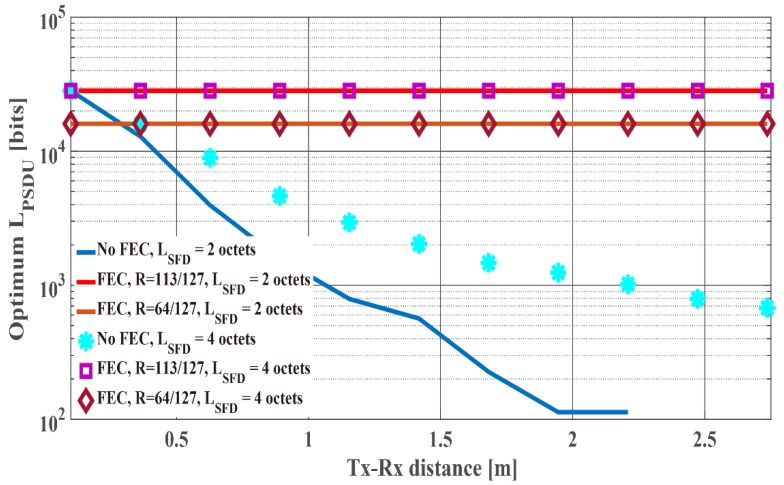
The optimum $L_{PSDU}$ under the IEEE model CM3 as a function of the Tx-Rx distance.

**Figure 24:**
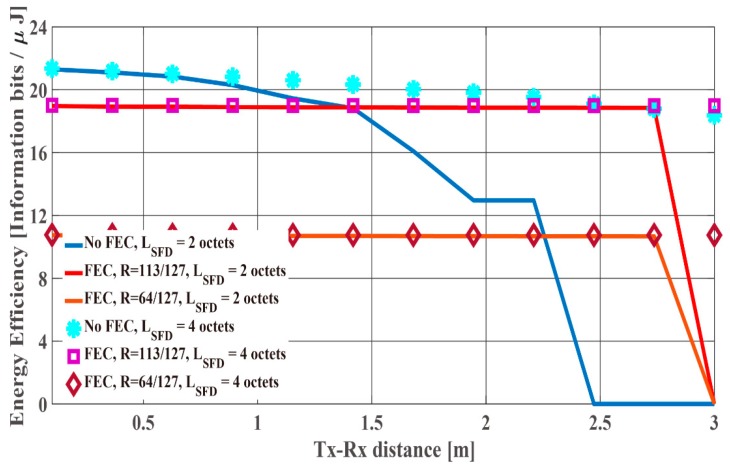
The maximum energy efficiency under the IEEE model CM3 as a function of the Tx-Rx distance.

Figure 23 and Figure 24 shows the optimum $L_{PSDU}$ and the maximum energy efficiency under the IEEE model CM3 as a function of the Tx-Rx distance. As shown in those figures, the optimum $L_{PSDU}$ and the maximum energy efficiency of the four-octet SFD are almost the same as those of the two-octet SFD in the cases of BCH encoding. On the other hand, the optimum $L_{PSDU}$ and the maximum energy efficiency of the four-octet SFD is much larger than those of the two-octet SFD in the no BCH encoding case. The reason is that $P_{fail,preamble}$ of the two-octet SFD is close to $10^{-2}$, and $P_{e,PSDU}$ of no BCH encoding case is higher than that of BCH encoding case. Hence, it can be said that the two-octet SFD is better in the short optimum $L_{PSDU}$ case, while the four-octet SFD is better in the long optimum $L_{PSDU}$ case from Figure 21, Figure 22, Figure 23 and Figure 24.

## 6. Conclusions

In this manuscript, we evaluated preamble detection in the ETSI SmartBAN PHY and proposed a modified preamble structure. Specifically, an SFD was added between the two-octet preamble and the PLCP header. The proposed preamble structure is compatible with the SmartBAN standard. This is because the general framework of the packet structure is not changed, and the only minor modification is made. Computer simulations indicated that the preamble with an SFD consisting of the four-octet orthogonal M-sequence has better detection performance than SmartBAN and similar approaches, in particular, under poor channel conditions with IEEE model CM3. In addition, integrated performance evaluation with respect to PER and energy efficiency considering preamble detection in SmartBAN PHY was conducted. According to computer simulation results, the case with an SFD whose $L_{SFD}=$ 4 octets and with an orthogonal M-sequence exhibited better PER performance, while larger energy efficiency was achieved in the case of an SFD whose $L_{SFD}=$ 2 octets and with an orthogonal M-sequence. Furthermore, it was possible to find the optimum $L_{PSDU}$ and the SFD satisfying the maximum energy efficiency with PER less than $10^{-2}$ from theoretical formulas. Those results suggested that it is better to change the length of SFD according to channel conditions and the optimum $L_{PSDU}$. For example, the two-octet SFD was better in case that the channel $E_{s}/N_{0}$ was high and the optimum $L_{PSDU}$ was short. On the other hand, the four-octet SFD was better in the long optimum $L_{PSDU}$ case.

In future research, other error-control schemes and access protocols will be evaluated and analyzed. In addition, the proposed system will be implemented in a real-world application. As the future prospects, we are aiming to amend the SmartBAN standard based on our proposed system. For example, it is conceivable to amend so that it can be selected whether or not to use the SFD, and the length of the SFD can be selected according to the node priority.

## Figures and Tables

**Table 1:** PHY throughput.

Symbol Rate (Mega-Symbol/Sec)	Coding Rate	$N_{R}$	Data Rate (Mbit/Sec)
1.0	1	1	1.0
1.0	1	2	0.5
1.0	1	4	0.25
1.0	113/127	1	0.89
1.0	113/127	2	0.44
1.0	113/127	4	0.22

**Table 2:** Computer simulation parameters.

Channel model	AWGN, IEEE model CM3
Path loss model	IEEE model CM3 (Hospital Room)
Frequency spectrum	2401 MHz–2481 MHz
Bandwidth (*BW*)	2 MHz
Modulation	GFSK
Bandwidth-time product (BT)	0.5
Modulation index (h)	0.5
FEC (PLCP Header)	(36, 22) shortened BCH code
FEC (PSDU)	(127, 113) BCH code
FEC (PSDU, proposed option)	(127, 64) BCH code
Maximum transmission power ($P_{tr}$)	0 dBm
Thermal noise density ($N_{0}$)	−174 dBm/Hz
Implementation losses (I)	5 dB
Receiver noise figure (NF)	10 dB
Information bit length ($L_{info}$)	678 bits
Preamble length ($L_{preamble}$)	2 octets
PLCP header length ($L_{PLCPheader}$)	40 bits
Symbol rate	1.0 Mega-symbol/sec
Inter-frame spacing duration ($T_{IFS}$)	150 μs

**Table 3:** SFD used in computer simulations.

Sequence Type		Bit Sequence (Hexadecimal)
Additional SFD 1		“01010101” (0 × 55)
Additional SFD 2		“10101011” (0 × AB)
Hadamard sequence. [32]	1 octet	“11001100” (0 × CC)
	2 octets	“1100001111000011” (0 × C3C3)
	4 octets	“11000011110000111100001111000011” (0 × C3C3C3C3)
Orthogonal M-sequence. [33]	1 octet	“11101000” (0 × E8)
	2 octets	“1111010110010000” (0 × F590)
	4 octets	“11111001101001000010101110110000” (0 × F9A42BB0)
Manchester- coded Orthogonal M-sequence. [33]	1 octet	“10100101” (0 × A5)
	2 octets	“1010100101100101” (0 × A965)
	4 octets	“10101010011001101001011001010101” (0 × AA669655)

**Table 4:** Each parameter of the IEEE model CM 3 path loss model.

	Hospital Room	Anechoic Chamber
*a*	6.6	29.3
*b*	36.1	−16.8
*σ_N_*	3.8	6.89

**Table 5:** Each parameter of the K factor of IEEE model CM 3.

$K_{0}$ [dB]	30.6
$m_{k}$ [dB]	0.43
$\sigma_{k}$	3.4

**Table 6:** $E_{s}/N_{0}$ when failure detection ratio satisfies $10^{-2}$ or less under the AWGN channel.

Sequence Type		$E_{s}/N_{0}\;[\mathrm{dB}]$
SmartBAN		16.6
Additional SFD 1		Not satisfied
Additional SFD 2		Not satisfied
Hadamard sequence	1 octet	14
	2 octets	6.0
	4 octets	3.0
Orthogonal M-sequence	1 octet	12.8
	2 octets	5.8
	4 octets	Less than 0
Manchester-coded Orthogonal M-sequence	1 octet	6.0
	2 octets	7.9
	4 octets	1.5

**Table 7:** $E_{s}/N_{0}$ when failure detection ratio satisfies $10^{-2}$ or less under IEEE model CM3.

Sequence type		$E_{s}/N_{0}\;[\mathrm{dB}]$
SmartBAN		Not satisfied
Additional SFD 1		Not satisfied
Additional SFD 2		Not satisfied
Hadamard sequence	1 octet	Not satisfied
	2 octets	5.5
	4 octets	Not satisfied
Orthogonal M-sequence	1 octet	Not satisfied
	2 octets	4.9
	4 octets	Less than 0
Manchester-coded Orthogonal M-sequence	1 octet	Not satisfied
	2 octets	Not satisfied
	4 octets	Not satisfied

**Table 8:** Communication distance when failure detection ratio satisfies $10^{-2}$ or less under IEEE model CM3.

Sequence Type		d [m]
SmartBAN		Not satisfied
Additional SFD 1		Not satisfied
Additional SFD 2		Not satisfied
Hadamard sequence	1 octet	Not satisfied
	2 octets	3.0
	4 octets	Not satisfied
Orthogonal M-sequence	1 octet	0.3
	2 octets	2.2
	4 octets	Over than 3
Manchester-coded Orthogonal M-sequence	1 octet	Not satisfied
	2 octets	Not satisfied
	4 octets	Not satisfied
